# Site-Specific Activity-Based Protein Profiling Using Phosphonate Handles

**DOI:** 10.1016/j.mcpro.2022.100455

**Published:** 2022-11-24

**Authors:** Wouter van Bergen, Johannes F. Hevler, Wei Wu, Marc P. Baggelaar, Albert J.R. Heck

**Affiliations:** 1 Biomolecular Mass Spectrometry and Proteomics, Bijvoet Center for Biomolecular Research and Utrecht Institute for Pharmaceutical Sciences, University of Utrecht, Utrecht, The Netherlands; 2 Netherlands Proteomics Center, Utrecht, The Netherlands; 3 Singapore Immunology Network (SigN), Agency for Science, Technology, and Research (A∗STAR), Singapore, Singapore; 4 Department of Pharmacy, National University of Singapore, Singapore, Singapore

**Keywords:** chemical proteomics, activity-based protein profiling, site-specific, inhibitor binding site identification, phosphonate affinity handles, Fe(III)-immobilized metal affinity chromatography, ABP, Activity-based probe, ABPP, Activity-based protein profiling, ACN, Acetonitrile, CuAAC, Cu(I)-catalyzed azide–alkyne cycloaddition, DPBS, Dulbecco's phosphate buffered saline, DTT, Dithiothreitol, EGFR, Epidermal growth factor receptor, ER, Endoplasmic reticulum, GLRX, Glutaredoxin-1, GSH, Glutathione, GSTP1, Glutathione S-transferase P, iBAQ, intensity-based absolute quantification, IMAC, Immobilized metal affinity chromatography, MQ, Milli Q, NCE, Normalized collision energy, PRDX, Peroxiredoxin, PSM, Peptide spectrum match, RTN4, Reticulon-4, TXN, Thioredoxin

## Abstract

Most drug molecules target proteins. Identification of the exact drug binding sites on these proteins is essential to understand and predict how drugs affect protein structure and function. To address this challenge, we developed a strategy that uses immobilized metal-affinity chromatography–enrichable phosphonate affinity tags, for efficient and selective enrichment of peptides bound to an activity-based probe, enabling the identification of the exact drug binding site. As a proof of concept, using this approach, termed PhosID–ABPP (activity-based protein profiling), over 500 unique binding sites were reproducibly identified of an alkynylated afatinib derivative (PF-06672131). As PhosID–ABPP is compatible with intact cell inhibitor treatment, we investigated the quantitative differences in approachable binding sites in intact cells and in lysates of the same cell line and observed and quantified substantial differences. Moreover, an alternative protease digestion approach was used to capture the previously reported binding site on the epidermal growth factor receptor, which turned out to remain elusive when using solely trypsin as protease. Overall, we find that PhosID–ABPP is highly complementary to biotin-based enrichment strategies in ABPP studies, with PhosID–ABPP providing the advantage of direct activity-based probe interaction site identification.

## Highlights

• IMAC-enrichable affinity tags (PhosID) are compatible with ABPP studies.
• PhosID in combination with ABPP (PhosID–ABPP) enables ABP binding site identification.
• PhosID–ABPP can be used in complex contexts, such as intact cells.
• We observe and quantify differences in binding sites in intact cells and cell lysates.
• Trypsin and pepsin provide complementary views of the ABP-interactome.

## Graphical Abstract

**Figure:**
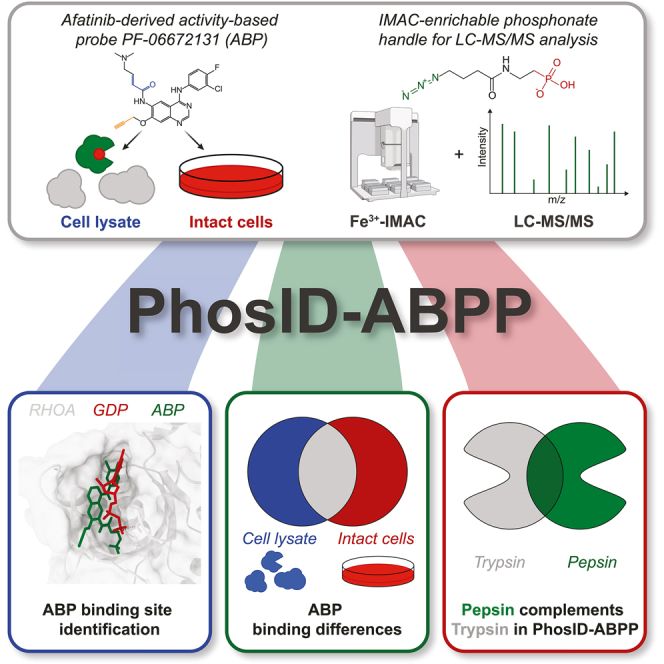

## In Brief

Here, we apply IMAC-enrichable phosphonate affinity tags for activity-based protein profiling, enabling the enrichment of peptides bound to the activity-based probe. Key advantages of PhosID–activity-based protein profiling are its high selectivity, efficiency and ease of use. PhosID–activity-based protein profiling allows the direct identification of the probe binding sites and is compatible with intact cell and cell lysate inhibitor treatment. Clear differences in binding sites were revealed in intact cells and in lysates of the same cell line.

Activity-based protein profiling (ABPP) can monitor targets and off-targets of small molecule drugs and is by now considered a powerful and versatile chemoproteomic strategy to advance drug discovery (1, 2). ABPP utilizes activity-based probes (ABPs) to interrogate activity or site occupancy status of proteins (1). The chemical probes used in ABPP generally consist of a ‘warhead’ to form a covalent bond with target proteins, a recognition element that enhances affinity for specific proteins, and a reporter tag that enables visualization or enrichment of targeted proteins (1, 3, 4, 5, 6, 7, 8, 9).

Protein–protein interactions, posttranslational modifications, and interactions with endogenous small molecules affect protein conformation and activity (10, 11, 12, 13). Therefore, it is crucial to monitor drug–protein interactions, ideally in the native protein environment. In contrast with many substrate-based enzyme assays, ABPP allows monitoring drug–protein interactions in complex cellular lysates, cell cultures, and can even be used in *in vivo* studies (1, 2, 14).

Because ABPs generally target a small fraction of the proteins in complex proteomes, enrichment strategies are typically required to identify and quantify ABP targets (1, 15, 16). Presently, biotin is by far the most popular affinity handle to retrieve ABP-targeted proteins. However, while strong streptavidin–biotin interactions facilitate stringent washing conditions to ensure very selective enrichment, elution from streptavidin is hampered, as harsh conditions are required for release (17, 18, 19). To circumvent these limitations, on-bead digestion of the bound protein facilitates the detection of the ABP-bound proteins. Unfortunately, this typically does not allow the specific detection of the exact ABP binding site, and ABP-bound protein identification is based on non-ABP–bound peptides, making these assignments somewhat ambiguous.

Identification of the exact ABP binding sites on proteins with an amino acid–specific resolution is desirable because (1) it will enhance the confidence in identifying *bona fide* ABP-targeted proteins; (2) it yields specific information on drug-protein interactions which can be used as restraints for structural modeling; and (3) specific site detection may reveal and distinguish multiple binding sites on the same protein. All in all, site-specific detection of ABPs is critical to improve drug development, as knowledge on binding mechanisms can be used to optimize drug binding and action.

To address issues with biotin for ABP binding site identification, various alternative approaches have already been explored to identify the ABP-labeled peptides, such as cleavable linkers and the use of desthiobiotin (18, 19, 20, 21). However, it still remains challenging to enrich and detect these low abundant ABP-bound peptides from a complex sample (21).

Recently, we reported strategies that employ phosphonate handles to enrich cross-linked peptides (Phox) and clickable phosphonate handles to enrich newly synthesized proteins (PhosID) (22, 23). This latter strategy avoids many of the limitations encountered with biotin, since the IMAC (immobilized metal-affinity chromatography) enrichment of chemically stable phosphonate-labeled peptides is highly efficient, readily automated, and facilitates easy release under mild conditions (22, 23). Encouraged by previous studies on direct ABP binding site identification and PhosID, we envisioned that the application of phosphonate-based clickable affinity handles could represent a powerful alternative strategy for ABPP, combining high sensitivity and efficiency with potentially unprecedented performance in exact binding site identification.

**Fig. 1:**
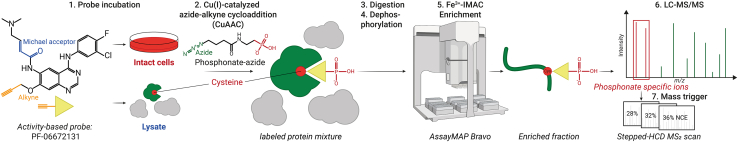
**Schematic representation of the here developed chemoproteomic site identification PhosID-ABPP strategy.** (1) Incubation of the activity-based probe (ABP) PF-06672131 in intact cells or in cell lysates. (2) Cell lysis and attachment of the phosphonate handle by Cu(I)-catalyzed azide–alkyne cycloaddition (CuAAC). (3) Subsequently, enzymatic digestion by trypsin or pepsin. (4) Dephosphorylation to remove endogenous phosphorylation. (5) Automated Fe^3+^-IMAC enrichment of ABP-labeled peptides. (6) Identification of ABP-labeled peptides that are separated, sequenced and identified by LC-MS/MS. (7) Mass triggering to ensure extensive fragmentation of ABP-labeled peptides. ABP, activity-based probe; ABPP, activity-based protein profiling; IMAC, immobilized metal-affinity chromatograph.

Here we report the application of phosphonate affinity handles for ABPP, enabling exact ABP binding site identification, which we term PhosID–ABPP (Fig. 1). In a proof-of-principal ABP study, we used the alkynylated afatinib derivative (PF-06672131), a cysteine reactive ABP known to target the ATP-binding pocket of the epidermal growth factor receptor (EGFR) (14). This probe has been employed in several biotin-based ABPP studies (14, 24) and revealed multiple off-targets for which the ABP binding sites were unknown.

## Experimental Procedures

### Synthesis of the Phosphonate-Azide

A stock solution of 500 mM 2-aminoethyl phosphonic acid (Sigma-Aldrich) was prepared in 1× Milli Q water (MQ, Millipore) and adjusted to pH 10 using sodium hydroxide. 75 μl of 0.5 M 2-aminoethyl phosphonic acid (Sigma-Aldrich) was incubated with 20 μl of 400 mM azidobutyric acid NHS (N-Hydroxysuccinimide) ester (Lumiprobe) in dimethylsulfoxide (Sigma-Aldrich) and 45 μl MQ. The reaction was incubated for at least 2 h at room temperature in the dark rotating, resulting in approximately 50 mM stocks of phosphonate-azide.

### Cell Culture

A431 cells (CRL-1555, ATCC) with a passage number below 20 were cultured in growth medium [(Dulbecco’s modified eagle medium (Lonza) supplemented with fetal bovine serum (HyClone GE) and 1× L-Glutamine (Lonza)]. Cells were kept in a humidified atmosphere with 5% CO_2_ at 37 °C in T175 flasks (Greiner). A431 cells were split twice a week by washing with Dulbecco’s phosphate buffered saline (DPBS, Lonza) and treatment with 0.05% Trypsin-EDTA (Gibco) for cell detachment. After detachment, trypsin was quenched by adding growth medium. 1/20 of the cell suspension was taken and grown with fresh growth medium in a new T175 flask.

### PF-06672131 Incubation in Intact Cells

5e^6^ A431 cells were plated in 15 cm plates (Greiner) 24 h before probe treatment and kept in a humidified atmosphere with 5% CO_2_ at 37 °C. The growth medium was replaced by treatment medium [growth medium with 25 μM PF-06672131 (Sigma-Aldrich)] and incubated at 37 °C, 5% CO_2_ for 4 h. Cells were detached with 0.25% Trypsin-EDTA (Gibco), and the cell suspension was spun down at 400 *g* for 5 min, and the supernatant was aspirated. The cell pellet was washed with DPBS before snap freezing the cell pellet in liquid nitrogen. The cell pellet was stored at −80 °C for later use.

### Cell Lysis

Cell pellets were lysed in 500 μl 1% sodium deoxycholate (Sigma) and 1× Protease inhibitor cocktail EDTA (Roche) in DPBS (Lonza) per 15 cm plate. The cells were suspended and incubated on ice for 30 min. Sonication was performed with a UP100H probe tip sonicator (Hielscher) using 60% amplitude with 1 s on and 1 s off for 1 min. Cell debris and DNA was spun down for 30 min at 20,567*g* at 16 °C. The supernatant was collected, and the protein concentration was determined by a bicinchoninic acid assay (Thermo Fisher Scientific).

### PF-06672131 Incubation in Cell Lysate

5.0 mg A431 cell lysates were treated with 100 μM PF-06672131 in 1 ml for 1 h at 37 °C. Methanol-chloroform precipitation was performed, and the air-dried pellets were resuspended in 500 μl 8 M urea (Sigma-Aldrich). The samples were sonicated with a bioruptor (Diagenode) for 10 min with 30 s on and 30 s off at high amplitude to fully dissolve all proteins.

### Sample Processing for SDS-PAGE

Cu(I)-catalyzed azide–alkyne cycloaddition (CuAAC) was performed on 30 μg protein lysates in 1× DPBS (pH 7.5) in 20 μl total volume. 2 μl mastermix of CuAAC components was added to a final concentrations of 15 μM of azide-fluor 488, 150 μM tris(3-hydroxypropyltriazolylmethyl)amine (Lumiprobe), 1.5 mM CuSO4 5·H2O (Sigma-Aldrich), and 1.5 mM tris(2-carboxyethyl)phosphine (Sigma-Aldrich). Samples were incubated for 2 h at room temperature while rotating. 200 mM dithiothreitol (DTT, Sigma) in 4× sample buffer XT (Bio-Rad) was added to reach a final concentration of 50 mM DTT and 1× sample buffer. The samples were boiled at 95 °C for 5 min. Afterward, the samples were cooled down to room temperature and loaded on a 4 to 12% bis-tris protein gel (Bio-Rad). Precision Plus Protein Dual Color Standards (Bio-Rad) was used as a molecular weight marker. After running, the gel was scanned with the “Cy2 channel” using an Amersham Imager 600 (GE Healthcare) to visualize the azide-fluor 488. Finally, Imperial blue protein stain (Thermo Fisher Scientific) was used to visualize the total protein loaded.

### Bioorthogonal Chemistry Reactions for Proteomics

CuAAC was performed on 5.0 mg protein lysates in 2 M urea (Merck) in 1× DPBS (pH 7.5). CuAAC components were added in the following order: 5 mM tris(3-hydroxypropyltriazolylmethyl)amine, 2.5 mM CuSO4 5·H2O, 500 μM phosphonate-azide (for preparation see “Synthesis of the phosphonate-azide”), and 25 mM sodium ascorbate (Sigma-Aldrich) in a final volume of 2 ml. Samples were incubated for 2 h at room temperature while rotating. Methanol–chloroform precipitation was performed to remove the CuAAC components, and the air-dried pellets were resuspended in 500 μl 8 M urea and sonicated in a bioruptor with high amplitude for 10 min with cycles of 30 s on and 30 s off.

### Sample Processing for Digestion

Clicked and dissolved protein samples were diluted to 4 M urea with 50 mM ammonium bicarbonate (pH 8, AmBic, Sigma-Aldrich). The proteins were reduced with 4 mM DTT (Sigma-Aldrich) for 60 min at room temperature and alkylated in the dark using 8 mM iodoacetamide (Sigma-Aldrich) for 30 min. Residual iodoacetamide was quenched by adding DTT to a final concentration of 4 mM. Next, samples were diluted 2× with 50 mM AmBic and digested with LysC (1:75 enzyme to protein ratio, Wako) for 4 h at 37 °C. Finally, proteins were digested overnight using trypsin (1:50, enzyme to protein ratio, Sigma-Aldrich) at 37 °C in a final volume of 2 ml. Digested material was desalted using 3 cc C18 Seppak cartridges (Waters) and air dried using a vacuum centrifuge.

For the digestion with pepsin, protease incubation (Porcine, 1:50, enzyme to protein ratio, Sigma-Aldrich) was performed for 4 h at 37 °C in 40 mM HCl in a total volume of 2 ml (pH 2). After incubation, pepsin was irreversible inactivated by adjusting the pH > 6 with 1M AmBic. Digested material was desalted using 3 cc C18 Seppak cartridges and air dried using a vacuum centrifuge.

### Dephosphorylation

Samples were dephosphorylated prior to IMAC enrichment. Desalted peptides were dissolved in 1 ml 1 × CutSmart buffer (New England BioLabs) and incubated with 50 units alkaline phosphatase (calf intestinal, QuickCIP, New England BioLabs) overnight at 37 °C while shaking. We previously showed that in contrast to normal phosphopeptides, the peptides modified with the probe-phosphonate handles are unaffected by phosphatase treatment (23). Following the dephosphorylation, all peptides were again desalted using 3 cc C18 Seppak cartridges (Waters) and air dried using a vacuum centrifuge.

### Automated Fe^3+^-IMAC Enrichment

Probe-phosphonate–labeled peptides were enriched using Fe(III)-NTA 5 μl (Agilent technologies) in an automated fashion by the AssayMAP Bravo Platform (Agilent Technologies). Fe(III)-NTA (nitrilotriacetic acid) cartridges were primed at a flow rate of 100 μl/min with 250 μl of priming buffer [0.1% TFA, 99.9% acetonitrile (ACN)] and equilibrated at a flow rate of 50 μl/min with 250 μl of loading buffer (0.1% TFA, 80% ACN). The flow through was collected into a separate plate. Dried peptides were dissolved in 200 μl of loading buffer and loaded at a flow rate of 5 μl/min onto the cartridge. Columns were washed with 250 μl of loading buffer at a flow rate of 20 μl/min, and the phosphonate-labeled peptides were eluted with 35 μl of ammonia (10%) at a flow rate of 5 μl/min directly into 35 μl of formic acid (10%). Flowthroughs and elutions were air dried afterwards and injected directly on a liquid chromatography-coupled mass spectrometer.

### LC-MS/MS

Samples were analyzed on a nanospray UHPLC system Ultimate3000 (Thermo Fisher Scientific) coupled to an Orbitrap Exploris 480 mass spectrometer (Thermo Fisher Scientific), in data-dependent acquisition mode. Peptides were trapped on an Acclaim Pepmap 100 C_18_ (5 mm × 0.3 mm, 5 μm) in solvent A (0.1% v/v formic acid in water) and then separated on an analytical column (Poroshell 120 EC C_18_, 50 cm × 75 μm, 2.7 μm, Agilent Technologies) with a flowrate of 300 nl/min. Elution fractions were measured with a gradient 9% solvent B (0.1% v/v formic acid in 80% ACN) for 1 min, 13 to 45% in 37 min, 45 to 99% in 3 min, and 99% solvent B for 4 min was used. Finally, the system was equilibrated back to 91% solvent A for 8 min. Periodic MS1 scans were performed at a resolution of 60,000, between 375 and 2000 m/z after reaching the normalized AGC target with automatic injection time every second. Top intense precursors were fragmented with normalized collision energy (NCE) of 28% and 12 s dynamic exclusion time in between two MS1 scans. HCD fragmentation was performed on precursors at a resolution of 30,000. Extra stepped-HCD scans with 28, 32, and 36% NCE were performed on a precursor each time 194.05822 or 247.08477 m/z ions were detected in the first MS2 scan, being signature fragment ions indicative of the ABP.

Total proteomes (before enrichment) were measured with a gradient of 9% solvent B for 1 min, 13 to 44% in 97 min, 44 to 99% in 3 min, and 99% solvent B for 4 min was used. Finally, the system was equilibrated back to 91% solvent A for 10 min. Periodic MS1 scans were performed at a resolution of 60,000, between 375 and 1600 m/z after reaching the normalized AGC target with automatic injection time every second. Top intense precursors were fragmented with NCE of 28% and 16 s dynamic exclusion time in between two MS1 scans.

### Database Search and Analysis

Trypsin-digested LC-MS/MS run files were processed using MaxQuant 2.0.1.0 or higher and the Andromeda search engine and searched against the human Swissprot database (version September 2020, 20,375 entries) (25). Enzyme specificity was set to Trypsin (C-terminal cleavage of lysine or arginine, except when proline follows) and up to three missed cleavages were allowed. Minimum peptide length was set to 4. Variable modifications of cysteine carbamidomethylation, methionine oxidation, carbamylation of lysines and N-termini, protein N-terminal acetylation and phosphorylation on serine, threonine ,and tyrosine were allowed, together with PF-06672131-phosphonate adduct on cysteine (C_29_H_34_N_9_O_6_ClFP; 689.20422). Diagnostic ions (194.05822, 247.08477, 690.21205 and 416.12896) and neutral losses (−274.08309, −292.09366, and −689.20422) were annotated as specific characteristic fragment ions for PF-06672131-phosphonate. Mass tolerance for precursors and fragment ions was 4.5 and 20 ppm, respectively. The integrated MaxQuant contaminants database was used to filter out for contaminants. A false discovery rate of 1% for peptide spectrum matches (PSMs) and proteins was allowed using a target-decoy approach. A score cutoff of 40 was used for modified peptides. For total proteome measurements, intensity-based absolute quantification (iBAQ) was enabled.

Pepsin-digested LC-MS/MS run files were searched against the human (20,375 entries) Swissprot database (version September 2020) using Fragpipe v18.0 with the MSFragger 3.5 and philosopher 4.3.0 search engine using the default settings (26). The integrated Fragpipe contaminant database was used for filtering out contaminants. Cleavage site was set to nonspecific and a peptide length between 6 and 30 was allowed. Oxidation of methionine, acetylation of the protein N terminus, and carbamidomethylation of cysteines were set as variable modifications. PF-06672131-phosphonate (689.20422) adduct was also set as a variable modification on cysteine. Precursor and fragment mass tolerance were set to 20 ppm both. False discovery rate for PSMs and proteins was set to 1% using a target-decoy approach.

### Statistical Analysis and Visualization

For MaxQuant output, the tables “evidence.txt” and “PF-06672131-phosphonateSites.txt” were used to generate a comprehensive table of the ABP binding sites and their relative abundance. Reverse and potential contaminants were filtered out. Intensities were used to calculate the relative abundance of modification in each sample. To calculate the relative abundance of proteins in A431 cells, the average iBAQ values were taken from the “proteinGroups.txt” table. For analysis of the ABP binding sites in IMAC elutions, only peptides modified with PF-06672131-phosphonate were kept for ABP binding site analysis. Peptides that were found in two out of three replicates were considered as ABP binding sites. Peptides with ambiguous localization of PF-06672131 (localization probability<0.75) were used for assessing the total numbers and intensities of PF-06672131-bound peptides, but not for site-specific analysis. Raw peptide intensities of ABP-bound peptides were log2 transformed. Data were checked for normal distribution before performing imputation of missing data with a downshift of 1.8 standard deviations and performing a Student’s *t* test. For samples digested with pepsin, the “psm.tsv” table was used for analysis, a minimal of two (peptide-spectrum match) PSMs per ABP binding site was accepted as a PF-06672131 binding site. Peptides with ambiguous localization of PF-06672131 (*i.e.*, peptides with multiple cysteines) were used for assessing the total number of PF-06672131-bound peptides, but not for site specific analysis. Analysis and visualization of data was done with Perseus 1.6.15, Excel 2016 and GraphPad Prism 9 (27). Venn diagrams were created using Biovenn (28). MS/MS spectra were visualized using in-house software, and figures were finalized in Adobe Illustrator.

### Experiment Design and Statistical Rationale

Lysate- and intact cell-treated samples combined with trypsin-based proteolysis were conducted with n = 3 biological replicates, enriched, and injected separately into the LC-MS/MS system. Each raw file was separately processed using the MaxQuant software. This number was sufficient to evaluate reproducibility and quantitatively compare the two conditions. Pepsin-based digestion was conducted with n = 1 replicate on an intact cell-treated sample, which is sufficient for proof of principle with an alternative protease without quantitative analysis.

### Protein-Ligand Docking

A crystal structure for RHOA bound to GDP (1FTN) was prepared for docking using PDB tools (29, 30, 31, 32, 33). A distance restraint of 1.7 to 1.9 Ångström between the sulfur on the targeted cysteine and the carbon on the probe was set as an unambiguous restraint. Residues (13–20, 33–37, 60–62, 118, 121, and 160–162) in the GDP-binding pocket were provided as ambiguous restraint, only used in the rigid body docking protocol. Docking was performed using the default protocol with minor adjustments for small molecule docking in HADDOCK 2.4 (32, 34). Rigid body docking was performed using 10,000 structures, and the best 400 structures were selected for semiflexible refinement according to ambiguous interaction restraints energies. The resulting 400 structures were analyzed and clustered according to RMSD with a cutoff of 2 and minimal cluster size of 4. The scoring for Evdw in the rigid body docking phase was set to 1.0 and the scoring for Eelec in the water refinement stage was set to 0.1. Moreover, number of molecular dynamics steps for rigid body and first rigid body cooling stage torsion angle molecular dynamics were set to 0. The initial temperature for second and third torsion angle molecular dynamics cooling and were set at 500 and 300 respectively. The best structure for RHOA was chosen based on the lowest HADDOCK scores, distance between the sulfur atom on the targeted Cys16 and the carbon atom on PF-06672131, and visual examination. Protein ligand interaction profiler web server was used to analyze the interactions between the probe and protein in the generated model (35). 3D models were visualized and exported using ChimeraX or PyMOL (36).

## Results and Discussion

### PF-06672131 Protein Labeling in Cell Lysates and Intact Cells Analyzed by in-Gel Fluorescence

To assess the efficiency of protein labeling by the probe, we first incubated an A431 (human skin cancer cell line) cell lysate and intact A431 cells with the ABP PF-06672131. After labeling, an azide-functionalized fluorescent reporter was introduced using the biorthogonal (CuAAC) reaction. In gel fluorescence revealed that the probe labeled many proteins in both cell lysates and intact cells, indicating that the used ABP displays a broad reactivity across the proteome (supplemental Fig. S1), as was reported previously (14). This reactivity pattern is similar in lysates and intact cells. However, we also observed differential labeling between cell lysate and intact cells (supplemental Fig. S1). Therefore, we next sought to identify the differences in binding sites of PF-06672131 in cell lysates and intact cells with PhosID-ABPP.

### Identification of Probe-Modified Peptides by MS/MS Fragmentation

**Fig. 2:**
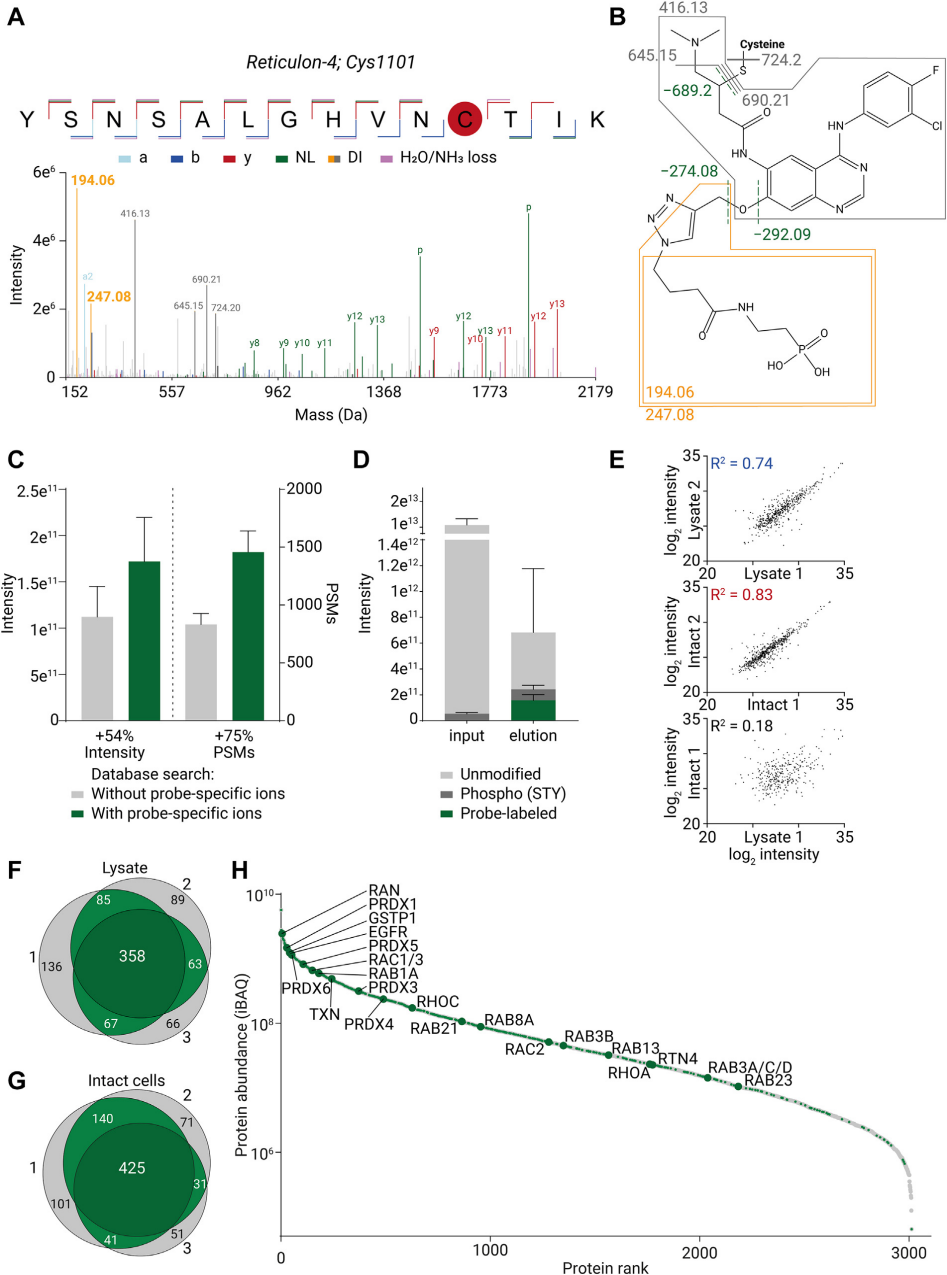
**Selective enrichment and identification of PF-06672131 binding sites.***A*, MS/MS spectrum of a PF-06672131-phosphonate-bound peptide originating from reticulon-4 (RTN4) labeled at position 1101. a: a-ion, b: b-ion, y: y-ion, NL: neutral loss, DI: diagnostic ion. *B*, fragmentation sites observed in the phosphonate-clicked ABP attached to peptides. Diagnostic ions originate from PF-06672131-phosphonate (194.06, 247.08, 416.13, 645.15, 690.21, and 724.20 *m/z*). The diagnostic ions with an *m/z* of 194.06 and 247.08 (*orange*) were used for mass triggering. The neutral losses −274.09, −292.09, −689.20 *m/z* are indicated in green. *C*, inclusion of neutral losses and diagnostic ions originating from the ABP-phosphonate modification in the MaxQuant database search boosts the identification of ABP-bound peptides. *D*, enrichment efficiency of intact cell-treated samples. Total MS1 intensity of peptides detected in the LC-MS/MS runs before (input) and after (elution) enrichment. *E*, correlation of the relative abundance of ABP-bound peptides detected by LC-MS/MS runs of lysate- and intact cell-treated samples. The correlation graphs of all individual replicates are provided in supplemental Fig. S3. *F*/*G*, Venn diagrams showing the overlap of detected ABP-labeled peptides between replicates in lysate (*F*), and intact cells (*G*). *H*, indication of ABP-labeled proteins (*green*) in an S-plot of proteins in A431 cells ranked by their relative abundance as estimated from their iBAQ values before enrichment. Proteins that are further discussed are indicated by the largest *dark green dots*. ABP, activity-based probe; iBAQ, intensity-based absolute quantification.

Detailed knowledge of the fragmentation pattern of ABP-bound peptides is crucial for their identification and detection, therefore initially manual examination of MS/MS spectra of ABP-bound peptides was conducted (37, 38). At first glance, when compared to unmodified peptides, the MS/MS spectra revealed multiple abundant nonannotated high-intensity peaks. We found that many of these initially nonannotated peaks in the MS/MS spectra originated from distinct neutral losses, namely 274.08 and 292.09 *m/z* (Fig. 2*A*), consistent with fragmentation at the ether bond (Fig. 2*C*). In addition, we observed the loss of the complete ABP-phosphonate (−689.20 *m/z*). Also, diagnostic ions at 194.06 *m/z* corresponding to fragmentation of the phosphonate moiety at the triazole ring could be robustly detected in the MS/MS spectra (Fig. 2, *A* and *B*), together with other PF-06672131-specific diagnostic ions (247.08, 416.13, 645.15, 690.21, and 724.20 *m/z*). Moreover, unreacted probe-phosphonate and fragments thereof are even observed at the MS1 level (690.21, 663.13, 645.15, and 416.13 *m/z*; supplemental Fig. S2*A*). By adding the neutral losses and the diagnostic ions in our MS/MS search strategy, we could increase the identification success of ABP-labeled peptides by 75% and increase the intensity of ABP-labeled peptides by 54% on average (Fig. 2*C*).

### Phosphonate Handles Enable Enrichment of Probe-Bound Peptides

As in most ABP experiments, peptides labeled by the ABP are relatively low in abundance compared to the background of the unmodified peptides. In our experiments, only 0.001% of the relative peptide abundance originated from ABP-labeled peptides (Figs. 2*D* and S2*C*). The low abundance of ABP-labeled peptides presents a significant additional challenge compared to the previously published PhosID method, in which the azidohomoalanine-labeled peptides constitute 0.2% of the total peptides quantified (23) or regular phosphoproteomics, in which phosphopeptides generally constitute 2 to 3% of the total peptide intensity before enrichment (39, 40, 41).

Consistent with the relative low abundance of ABP-labeled peptides, we identified, without using any enrichment, only two PSMs for probe-labeled peptides in A431 cell lysates. In contrast, by using the efficient automated phosphorylated peptide enrichment on an Assaymap BRAVO system using High-Capacity Fe(III)-NTA Cartridges, we could increase the relative abundance of ABP-labeled peptides on average to about 23%, identifying around 1500 PSMs (*i.e.,* a 750-fold increase in PSMs). After the IMAC-based enrichment, the remaining 77% of intensity constituted of phosphopeptides (14%) and unmodified peptides (63%) (Figs. 2*D* and S2, *C* and *D*). On the MS1 chromatography trace, the change in sample constitution before and after enrichment shows a depletion of a majority of the (unmodified) peptides (supplemental Fig. S2*A*).

The use of the probe-related low mass diagnostic ions in the MS/MS spectra turned out to be a powerful tool to increase the sensitivity of our method (42). Therefore, in addition to the offline phosphopeptide enrichment, we used a mass triggered method to increase the sensitivity aiming to identify also lower abundant probe-labeled peptides. We therefore used probe-specific diagnostic fragment ions of 194.06 and 247.08 *m/z*.

### PhosID–ABPP Reproducibly Identifies ABP Binding Sites in Either Lysates or Intact Cells

Using this optimized strategy, we were able to detect more than 500 ABP binding sites in at least two out of the three experiments performed on A431 lysates (Fig. 2*F* and supplemental Data S1). To investigate if we could extend our strategy to detect ABP binding sites in intact cells, we incubated intact A431 cells with PF-06672131. Our method performed also very well when applied to intact cells, and we reproducibly identified, under these conditions, around 600 ABP binding sites (Fig. 2*G* and supplemental Data S1). Interestingly, we also found that the ABP labeled multiple unique binding sites in many proteins. (supplemental Fig. S2*E*). To validate if the ABP binding site detection strategy does not affect the ABP target scope, we compared the protein targets that were detected earlier by Lanning *et al.* who used a biotin-based protein-centric ABPP approach to find PF-06672131 protein targets (14). Three hundred two of the 437 proteins that we identified in intact A431 cells were also found by Lanning *et al.* by intact cell treatment with PF-06672131. Additionally, we observed 43 probe binding sites on 25 kinases, of which 11 were also detected by Lanning *et al.* The high overlap indicates that the ABP binding sites that we identify by PhosID–ABPP are within proteins that are enriched by using the earlier described biotin-based affinity approach (14). From this, we conclude that the PhosID–ABPP approach does not seriously affect the target landscape of the ABP.

To assess the relative abundance of ABP-targeted proteins in the A431 cells, we assessed the protein abundance in A431 cells as determined by iBAQ in the LC-MS measurements before Fe^3+^-IMAC enrichment (Fig. 2*H* and supplemental Data S2). This comparison revealed that many abundant proteins within the proteome were found to interact with the probe, which may be caused by, in contrast with less abundant proteins, low labeling stoichiometry of abundant proteins being already sufficient for ABP-labeling site detection. Importantly, PhosID–ABPP was found to detect binding sites on proteins ranging deep into the lower abundant regions of the proteome (Fig. 2*H*).

### PhosID–ABPP Exposes Protein and Site-Specific Differences in Protein Binding When Applied to Lysates or Intact Cells

**Fig. 3:**
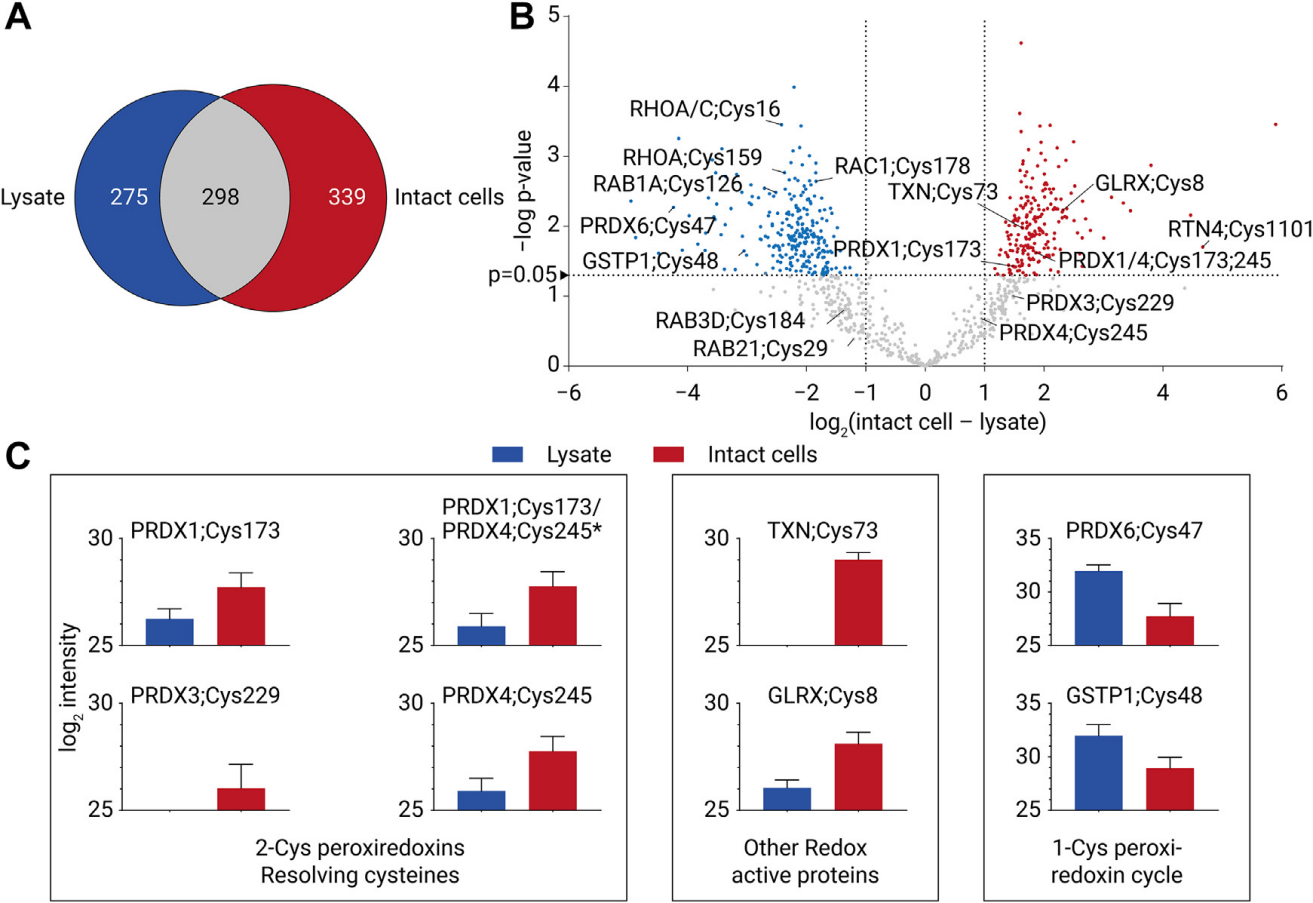
**Substantial differences observed in ABP-labeling of cell lysates and intact cells**. *A*, Venn diagram showing an overlap of the PF-06672131-bound peptides detected in intact cells (*red*) and cell lysates (*blue*). *B*, volcano plot showing the difference in labeling intensity through a *t* test between lysates and intact cells. ABP binding sites highlighted in *red* are >2-fold significantly enriched in intact cells compared to lysates, ABP binding sites highlighted in *blue* are >2-fold significantly enriched in cell lysates compared to intact cells. *C*, PF-06672131 binds catalytic cysteines on redox-sensitive proteins. Log_2_ intensities from PF-06672131-bound peptides derived from redox proteins in lysates (*blue*) and intact cells (*red*). ∗ Shared tryptic peptide (HGEVCPAGWK) around the peroxidatic cysteine on PRDX1 and PRDX4 was detected next to the unique peptides for PRDX1;Cys173 (HGEVCPAGWKPGSDTIKPDVQK) and PRDX4;Cys245 (HGEVCPAGWKPGSETIIPDAGK). ABP, activity-based probe; PRDX, peroxiredoxin.

We observed remarkable differences in ABP labeling between intact cells and cell lysates (Fig. 3*B*). Nonetheless, the correlation between the intensities of ABP-bound peptides in replicate experiments was found to be high, both for lysate-labeled replicates (R^2^ >0.7) or intact cell-labeled replicates (R^2^ >0.8). However, the correlation between the intensities of ABP-bound peptides intact cells and cell lysates was consistently found to be below an R^2^ of 0.3 (Figs. 2*E* and S3). Moreover, a Venn diagram and statistical analysis of the PF-06672131 binding sites observed in the intact cells and the cell lysates showed that there exists an overlap in binding sites, but also a substantial difference in ABP binding sites between lysates and intact cells (Fig. 3, *A* and *B* and supplemental Data S3). The observed difference might be affected by using two different ABP concentrations in cell lysates (25 μM) and intact cells (100 μM) and may also be influenced by the lysis conditions. However, these factors do likely not explain the stark contrast in ABP labeling efficiencies between cell lysates and intact cells for specific sites. To gain insight into the origin of these differences, we focused on some of the most pronounced site-specific differences observed in a subset of ABP-targeted proteins.

### Reticulon-4 Is a Top Binding Target of the ABP in Intact Cells

The labeling of a target protein depends on both the specific reactivity of the probe toward that site/protein and the abundance of this protein in the proteome. Consequently, proteins that are low in abundance in the proteome but high in abundance in our data set of probe-labeled peptides are likely earnest targets of the probe. Log_2_ ratios of ABP-peptide intensity over iBAQ values were calculated and used as an approach to prioritize ABP target sites (supplemental Data S1). A peptide containing probe-labeled Cys1101 of reticulon-4 (RTN4) belongs to the top 10 most intense ABP-labeled peptides in our data on the intact cells. Interestingly, this peptide is labeled around 55-fold more intense in intact cells compared to cell lysates. RTN4 has a relatively low abundance in A431 cells (iBAQ: 4e^7^, log_2_(probe-peptide intensity/iBAQ): 5.44, Figs. 2*H* and 3*B*, supplemental Datas S1, S2, and S3). RTN4 is thought to be located in the endoplasmic reticulum (ER) and plays an important role in maintaining the ER and the formation of ER tubules (43). RTN4 is emerging as a promising target in cancer therapy, and targeting of Cys1101 by the covalent inhibitor DKM 3-30 has been reported to result in aberrant ER tubule formation and mitosis, reducing tumor cell growth (43, 44, 45). The targeted cysteine is hypothesized to be exposed on the cytosolic side of the ER membrane, making it readily accessible for covalent labeling (44). Reduced labeling in cell lysates, as observed clearly in our data, may be caused by conformational changes resulting from ER membrane disruption or oxidative modification of the Cys1101 upon cell lysis.

### Substantial Differences in Lysate and Intact Cell Labeling Relate to Redox-Sensitive Cysteines on Proteins Involved in Redox Signaling

We observed that several functional cysteines from proteins involved in redox signaling were labeled by the ABP very distinctively in the cell lysates compared to intact cells (supplemental Data S1) (Fig. 3*C*). Among these proteins were found to be several members of the peroxiredoxin (PRDX) family (PRDX1, PRDX3, PRDX4, and PRDX6), thioredoxin (TXN), glutaredoxin-1 (GLRX), and glutathione *S*-transferase P (GSTP1) (46). Three PRDX proteins, PRDX1, PRDX3, and PRDX4, detected are 2-Cys PRDXs that reduce H_2_O_2_ by donating an electron from the peroxidatic cysteine (47). The carboxy-terminal cysteine from another 2-cys PRDX can consecutively form a disulfide bond to generate a PRDX-dimer. In PRDX1, PRDX3, and PRDX4, the conserved carboxy-terminal-resolving cysteines are Cys173, Cys229, and Cys245, respectively. TXN is involved in reducing this disulfide bond (47). Whether this carboxy-terminal–resolving cysteine of PRDX is free or involved in a disulfide bridge to form a dimer depends on various factors, such as concentrations of H_2_O_2_ and TXN (47). Consistently in all our ABP labeling replicates, the carboxy-terminal cysteines of PRDX1, PRDX3, and PRDX4 are found to be intensely labeled in intact cells, and either not detected or low-level labeled in the cell lysates, indicating that these free cysteines become oxidized and substantially less available for ABP labeling in the cell lysates (Fig. 3*C*).

While we observe similar probe-labeling intensities for peroxidatic Cys47 in 1-cys PRDX6 in intact cells, a loss or decrease of signal of this site in lysates is not observed. On the contrary, Cys47 was more abundantly labeled in lysate compared to intact cells. Cys48 on GSTP1, a partner for heterodimerization with PRDX6, which acts as the resolving cysteine for PRDX6, shows a similar trend (48, 49). GSTP1 heterodimerizes with oxidized PRDX6 and catalyzes the *S*-glutathionylation of Cys47, during which a disulfide bridge between Cys47 on PRDX6 and Cys48 on GSTP1 can occur (48). To acquire a reduced PRDX6, PRDX6-GSH (glutathione) interacts with another GSH molecule to form oxidized GSH (47, 50). More intense labeling in lysate by the ABP might indicate that PRDX6 and GSTP1 are not oxidized upon lysis, unlike the 2-cys PRDXs and could thus be probe targeted.

TXN, is abundantly labeled on Cys73, again only in intact cells. Cys73 is involved in the formation of TXN homodimers and thereby regulates TXN activity (51). In addition to dimer formation, the activity of TXN can also be regulated by *S*-nitrosylation or *S*-glutathiolation of Cys73 (52, 53). Moreover, PX-12, a TXN inhibitor, is also proposed to exert its effect through binding at Cys73 (54). The abundant ABP binding of TXN Cys73 in intact cells and the lack of labeling in cell lysates could again be due to the free cysteine being oxidized or modified differently upon formation of the lysate (Fig. 3*C*).

Finally, in GLRX, we observe Cys8 to be abundantly ABP labeled in intact cells in all intact cell replicates, but not in cell lysates. GLRX catalyzes deglutathionylation through the nucleophilic displacement of the GSH moiety by the active site cysteine, followed by the rate-limiting step where the thiolate ion of the active site cysteine is regenerated consuming one molecule of GSH (55). Cys8 has been linked to oxidative inactivation of GLRX and has also been reported to potentially be modified *via S*-nitrosylation (56). The abundant ABP binding of GLRX Cys8 in intact cells and the lack of labeling in cell lysates could again be due to the free Cys8 becoming rapidly modified upon formation of the lysate (Fig. 3*C*).

### PF-06672131 Is Directed Toward Nucleotide-Binding Pockets

**Fig. 4:**
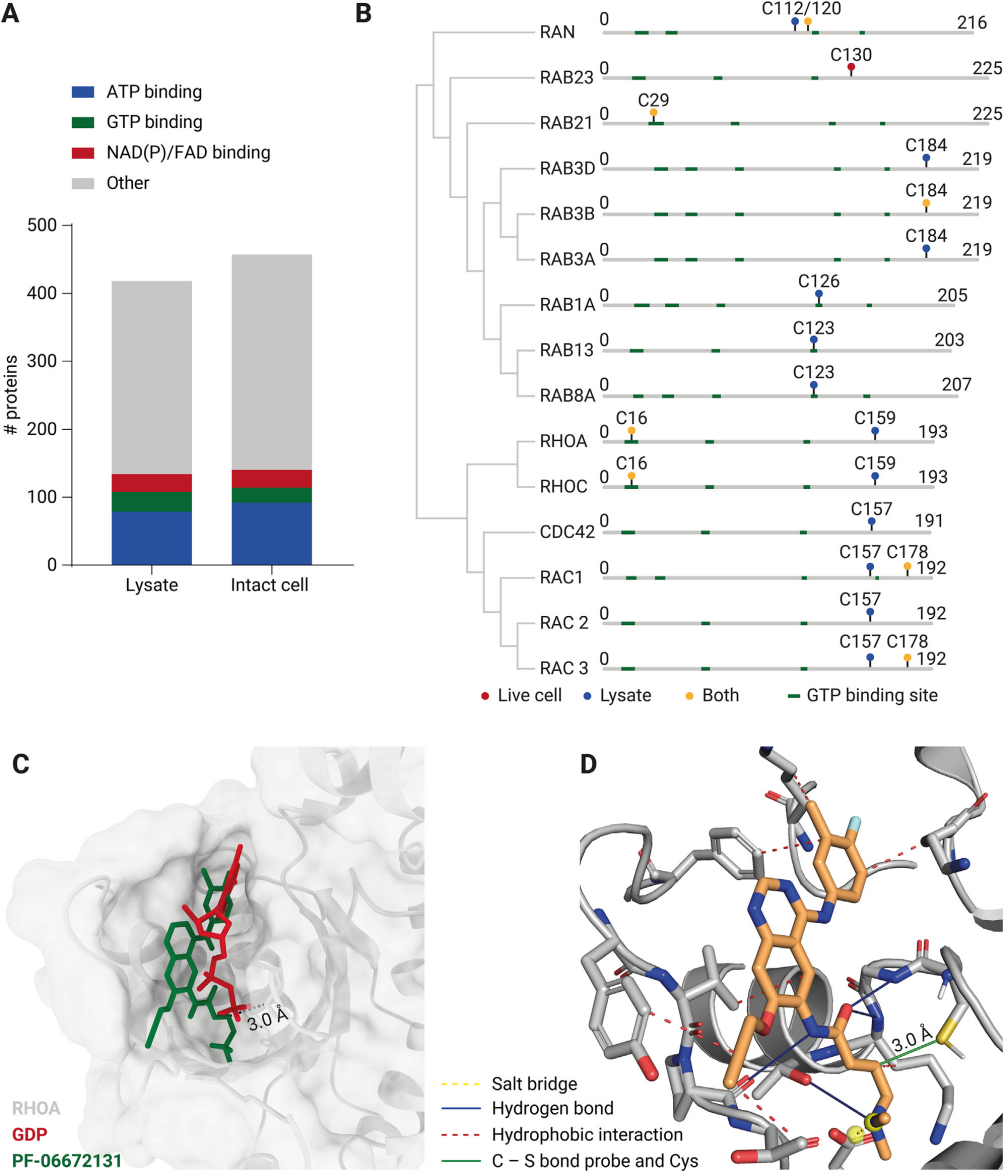
**PF-06672131 binds nucleotide-binding proteins, including the Ras superfamily**. *A*, bar graph of the number of different nucleotide (ATP, GTP, NAD(P), FAD)-binding proteins detected to be probe-bound in cell lysates and intact cells, about 30% of the ABP-bound proteins could be classified as nucleotide binding proteins (PANTHER database (58, 72)). *B*, cysteine residues found to be reacting with PF-06672131 of proteins belonging to the Ras superfamily. *C*, docking of PF-06672131 onto Cys16 in the GDP/GTP-binding site in a crystal structure of RHOA (PDB: 1FTN) (33). PF-06672131 is indicated in *green* and GDP is indicated in *red*. *D*, Protein ligand interaction profiler web server analysis reveals that next to the covalent bond to cysteine (*green line*), PF-06672131 (*orange*) might interact with RHOA (*gray*) through four hydrogen bonds (*blue lines*), seven hydrophobic interactions (*red dashed lines*), and one salt bridge (*yellow dashed line*) (supplemental Table S1) (35). ABP, activity-based probe.

Afatinib and its derived ABP PF-06672131 have been shown to bind in an ATP-binding pocket of EGFR (14, 29). Therefore, it has been hypothesized that PF-06672131 mimics ATP and may also display affinity for ATP-binding pockets belonging to proteins other than EGFR (14, 24). We indeed observed that around 20% of the detected probe-targeted proteins are ATP binders, which corresponds to a 2.5-fold enrichment compared to the percentage of ATP binders in the total human proteome as determined by GO term molecular function overrepresentation analysis (PANTHER, Protein analysis through evolutionary relationships) (57, 58). In addition to ATP binders, around 10% of the probe targets are known to bind other nucleotides, including GTP (6%) and NAD (3%) (Fig. 4*A*). Together, these observations point to a preferential targeting of PF-06672131 toward ATP- and other nucleotide-binding proteins.

### PF-06672131 Binds to Conserved Cysteines in GTP-Binding Pockets From the Ras Superfamily of GTPases

Ras super family proteins are involved in targeting and regulation of vesicular membrane trafficking (59, 60). These small GTPase proteins act as molecular switches that are turned on by guanosine exchange factors that catalyze the conversion of the GDP- to the GTP-bound state and are ‘switched off’ by GTPase-activating proteins, which enhance GTP hydrolysis to GDP. In their active GTP-bound state, Ras super family proteins recruit effector proteins through which they exert their biological effects (59, 61). Since the employed ABP is specifically directed toward cysteine residues in proteins, it is important to note that besides regulation by GTPase-activating proteins and guanosine exchange factors, small GTPases are also highly and tightly regulated by various cysteine posttranslational modifications, including farnesylation, *S*-palmitoylation, glutathionylation, and disulfide bridge formation (62, 63, 64, 65, 66).

The nuclear small GTPase RAN was found to contain two ABP labeling sites within the same tryptic peptide, on Cys112 and Cys120 (Fig. 4*B* and supplemental Data S1). Nevertheless, localization and intensity of probe-binding could be distinguished, and it was revealed that Cys120 was found to be more intensely labeled in lysate and in intact cells (supplemental Data S1). A previous study hypothesized that Cys112 is an oxidation target by pervanadate and causes degradation of RAN (67). Cys120, on the other hand, was shown not to be a target of pervanadate-mediated oxidation. Cys120 being more labeled in both conditions might indicate that Cys112 is more frequently oxidized and therefore less available for ABP binding in both lysate and intact cells.

Within the Rab small GTPase protein family, the ABP targets a conserved cysteine in the nucleotide-binding site of RAB1A (Cys126), RAB8A (Cys123), and RAB13 (Cys123), which are all labeled substantially more in cell lysates compared to intact cells, consistent with the hypothesis of reduced competition with endogenous nucleotides due to lower local nucleotide concentrations in lysates compared to intact cells (68). The observed probe-labeled cysteines in RAB3A/D (Cys184) and RAB3B (Cys184) are not situated in the GTP-binding pocket but are surface exposed and known to be next to the complementary determining region 3 which plays a role in the binding of the small GTPase to its effectors (66). In a co-crystal structure of *Rattus norvegicus* RAB3A bound to its effector protein Rabphilin (PDB:1ZBD), Cys184 is positioned at the binding interface of the two proteins (69). Therefore binding of Cys184 by the ABP could potentially also block the interaction between RAB3A and Rabphilin.

The Rho GTPase family forms part of the Ras superfamily that regulate a wide range of cellular responses, including cell adhesion and changes to the cytoskeleton (64). Multiple members of this subfamily are also labeled at the conserved cysteine residues Cys159 in RHOA, RHOC, and Cys157 in CDC42, within their nucleotide-binding pockets. RAC1, RAC2, and RAC3 appear to also be targeted at Cys157. However, the relative labeling efficiency between the isoforms RAC1, RAC2 and RAC3 cannot be determined as they share identical Cys157-containing tryptic peptides. These cysteines all show preferential labeling in the cell lysate, which again, is in line with lower concentrations of competing nucleotides in cell lysates. Cys157 in RAC1 is known to be regulated by glutathionylation during metabolic stress, which is proposed to have an inhibitory effect on RAC1 activity (70).

Moreover, other cysteines in the Rho GTPase family that undergo posttranslational modifications are targeted by the ABP. For example Cys16 of RHOA, which is in close proximity of Cys157 in the GTP-binding pocket, can inactivate the protein by formation of a disulfide bond with another cysteine (Cys20) (65). Probe labeling of this specific Cys16 was identified in a tryptic peptide that is shared by RHOA and RHOC in both cell lysates and intact cells. In addition, we identified labeling of Cys178 on a tryptic peptide shared by RAC1 and RAC3, this cysteine is a known target for *S*-palmitoylation which regulates protein localization and affects GTP binding (71).

Together these probe interactions reveal that PF-06672131 targets multiple sites in the Ras superfamily including sites residing in nucleotide-binding pockets and sites that undergo posttranslational modifications. Knowledge of the exact ABP binding site can guide predictions of the functional effect of inhibitor treatment for specific proteins. In addition, the observations in the Rho family uncover the limitation that the exact identity of a protein target cannot always be unequivocally determined based on single peptides, especially for proteins with close homologs. This limitation might however be resolved by using other proteases in addition to trypsin to generate unique peptides.

### In Silico Binding Pose Prediction in RHOA

To simulate the interaction between PF-06672131 and RHOA, HADDOCK 2.4 was used to dock the ABP on Cys16 in the GDP/GTP-binding pocket on a RHOA crystal structure (PDB: 1FTN) (32, 33, 34). The best-fitting docking pose with the lowest distance of the carbon of PF-06672131 to the Cys16 sulfur atom shows that the ABP fits well in the RHOA nucleotide-binding pocket (Fig. 4*C*). A distance of 3.0 Ångström for the sulfur-carbon bond between the ABP and Cys16 of the protein was measured. Given the resolution of 2.2 Ångström and a C – S bond being 1.8 Ångström, this falls within the error margin. Additional analysis of the interactions between PF-06672131 and RHOA shows that the probe might bind in the GTP-binding pocket through four hydrogen bonds, seven hydrophobic interactions and a salt bridge, hinting that affinity of the probe for the GTP-binding pocket may partially originate from these interactions (Fig. 4*D* and supplemental Data S1) (35). The *in silico* binding pose of the ABP in RHOA indicates that computational efforts guided by our mass spectrometry data may gain insight of inhibitor binding poses. Knowledge on the exact binding site of the ABP significantly limits the possible binding poses, increasing the accuracy of the model. Therefore, ABPP–PhosID in combination with computational modeling can contribute to drug development by guiding inhibitor optimization.

### Pepsin Digestion Allows Detection of PF-06672131 Binding to Noncatalytic Active Site Cysteine in EGFR

**Fig. 5:**
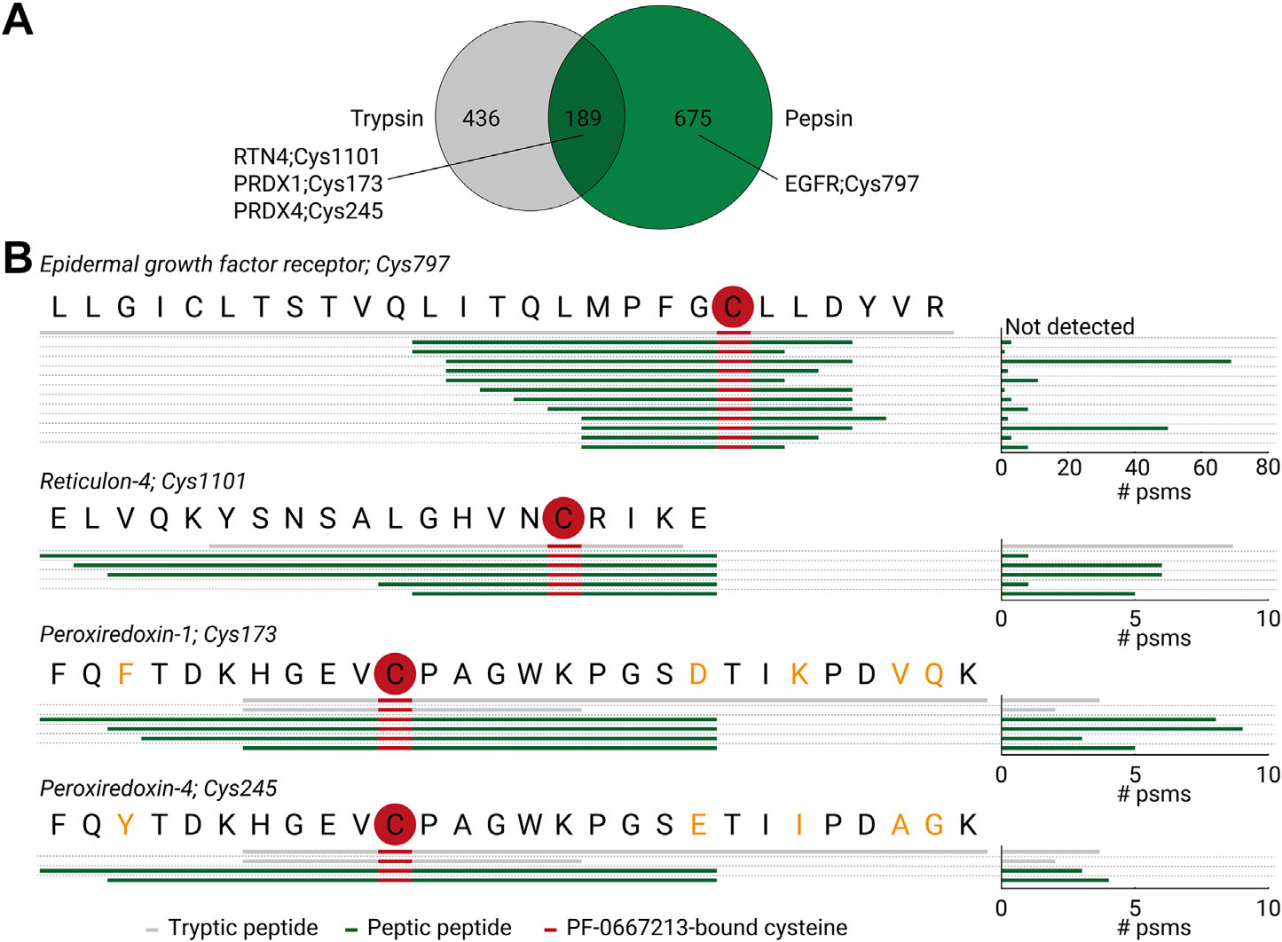
**Proteolysis with pepsin widens the landscape of identified ABPbinding sites, including the site on EGFR**. *A*, Venn diagram indicating the overlap of ABP binding sites found by trypsin (*gray*) and pepsin proteolysis (*green*) of probe-treated intact cells (supplemental Data S4). Pepsin expands the scope of PhosID-ABPP, with 675 sites exclusively detected with the pepsin-based approach, including the supposed primary binding target and site of PF-06672131 on EGFR Cys797. 189 probe-targeted cysteines were found in common when using trypsin and pepsin. *B*, number of psms found for evidence of individual ABP-bound peptides. Peptides with ABP binding site Cys797 on EGFR, Cys1101 on RTN4, Cys173 on PRDX1, and Cys245 on PRDX4 through a trypsin (*gray*) and pepsin (*green*) approach are shown. The *red* cysteines indicate the detected binding site of PF-06672131. The *orange* annotated amino acid letters indicate difference in sequence between PRDX1 and PRDX4. ABP, activity-based probe; ABPP, activity-based protein profiling; EGFR, epidermal growth factor receptor; PRDX, peroxiredoxin.

Initially, we did not detect binding of PF-06672131 to its known target, Cys797 in the ATP-binding pocket of EGFR. We hypothesized that the size and the hydrophobic nature of the generated tryptic peptide might hamper detection using our proteomics LC-MS/MS methodology (27 amino acids, Grand average of hydropathy, GRAVY: 1.19, Fig. 5*B*). Therefore, we did explore other proteases for the digestion and found especially pepsin very useful. Using pepsin as protease, probe-labeled Cys797 containing peptides were abundantly detected in intact cells with 161 PSMs, originating from 12 different peptides (Fig. 5*B* and supplemental Data S4). This high amount of different pepsin-derived peptides can be explained by the more diverse cleavage specificity of pepsin compared to trypsin. Comparing the target landscape between pepsin- and trypsin-mediated PhosID–ABPP revealed that the observed overlap in probe-bound cysteines is relatively low (189 sites), and the pepsin-based approach revealed an extra 675 ABP binding sites (Fig. 5*A*). Thus, strategies using alternative proteases can strengthen PhosID–ABPP by expanding the landscape of detected binding proteins and their binding sites. Moreover, the evidence for specific probe binding sites can be improved using two or more proteases to generate multiple peptides containing the site of ABP–protein interaction, *e.g.*, Cys1101 on RTN4 and Cys173 on peroxiredoxin-1 (Fig. 5*B*). Differential enzymatic digestion also provides the opportunity to distinguish probe-bound cysteines between variants of proteins with high homology. For example, PRDX1 and PRDX4 share high homologous regions around the active site cysteines Cys173 and Cys245, respectively, and could not be discerned with the tryptic peptide (HGEVCPAGWK, Fig. 5*B*). Using pepsin, these variants could be distinguished with peptides that are unique for PRDX1 or PRDX4, indicating that Cys173 on PRDX1 was more intensely labeled than Cys245 on PRDX4, as the total number of PSMs for Cys173 on PRDX1 was found to be higher (Fig. 5*B*). Together, these data show that alternative protease strategies strengthen PhosID–ABPP by revealing novel binding sites, improving the evidence for ABP binding sites through different enzymatic cleavages and distinguish ABP binding sites on different proteins sharing high homology.

## Conclusion

Here we introduce a new ABPP approach based on employing IMAC-enrichable phosphonate affinity tags, allowing the efficient and selective enrichment of peptides bound to an ABP, with as clear benefit enabling the identification of the exact drug binding site. Using PhosID–ABPP, we were able to robustly and reproducibly detect hundreds of protein targets, charting the site-specific target landscape of the alkynylated afatinib derivative ABP (PF-06672131) in human A431 skin cancer cells. The exact binding site profile revealed that the ABP not only reacted with active site cysteines in the ATP-binding pocket of EGFR but also to many surface accessible cysteines and cysteines in binding pockets of not only ATP but also other nucleotides. PhosID–ABPP was applied to both intact cells and cellular lysates. Many differences in labeling of specific cysteines were observed between intact cells and cell lysates. Some of these differences may originate from redox-sensitive cysteines undergoing oxidation–reduction cycles during protein function and differences in nucleotide-binding status of proteins in lysates *versus* intact cells. Through the use of pepsin as an alternative protease, instead of the commonly used trypsin, we detected the main EGFR target binding site of PF-06672131 with multiple different peptides, demonstrating that the utility of PhosID-ABPP could be further augmented by orthogonal use of proteolytic enzymes.

PhosID-ABPP allows future studies on site-specific drug occupancy by concentration- and time-dependent competition with the parent inhibitor Afatinib, and we anticipate that the workflow can be readily extended to other ABPs. With this, we envision PhosID-ABPP as a highly valuable addition to the ABPP toolbox, which is also complementary to existing protein-level enrichment strategies.

## Data Availability

The mass spectrometry proteomics data have been deposited to the ProteomeXchange Consortium *via* the PRIDE partner repository with the dataset identifier PXD036569. This article contains supplemental data: Supplementary Figures 1-3, Supplementary Table 1, and Supplementary Data Excel files 1 to 4.

## Supplemental data

This article contains supplemental data (20).

## Conflict of interest

The authors declare no competing interest.
