# Supplementary material for: Site-Specific Activity-Based Protein Profiling Using Phosphonate Handles
Source: Mol Cell Proteomics. 2022 Nov 24;22(1):100455. doi: 10.1016/j.mcpro.2022.100455 (PMC9803953; doi:10.1016/j.mcpro.2022.100455)
Supplement: Supplemental Figures and Tables [file mmc5.docx]

*
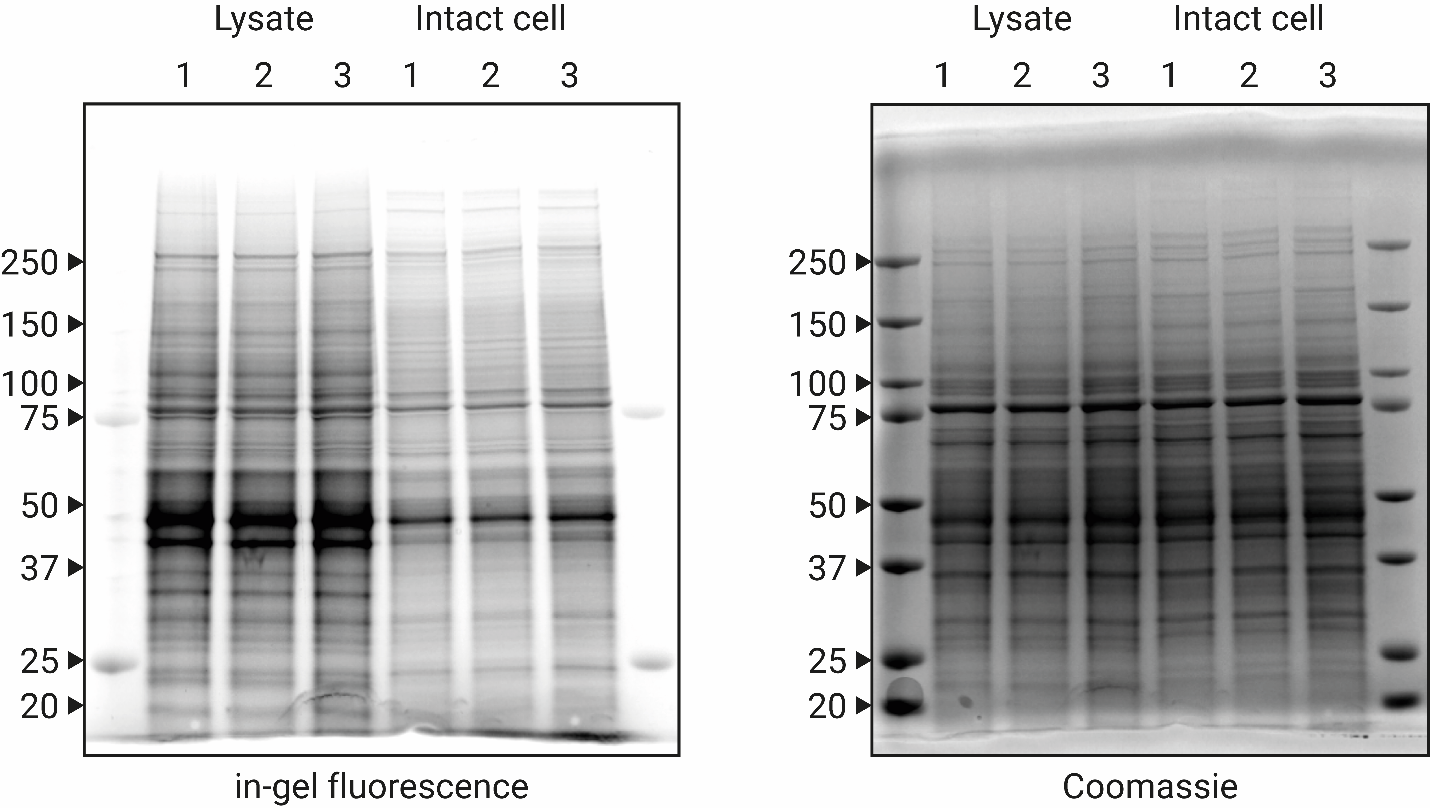
*

**Supplementary Figure 1:** On the left, in-gel fluorescence detection of PF-06672131-binding proteins in cell lysates (first three lanes) and intact cells (lane 4 to 6). On the right, total Coomassie stained gels for estimating equal amounts of total protein loaded on SDS-PAGE.


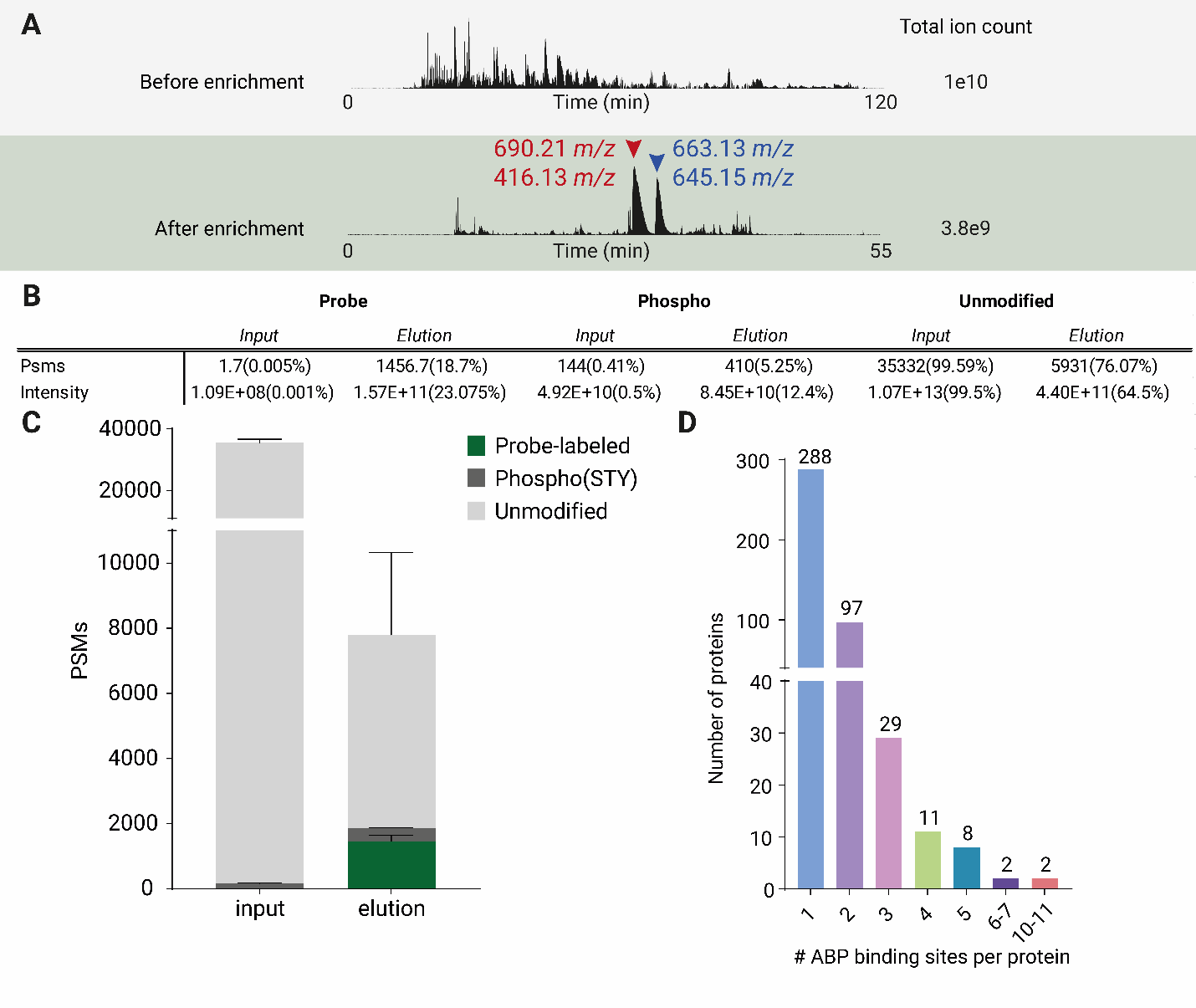


**Supplementary Figure 2**: Enrichment of ABP bound peptides by using PhosID-ABPP. **A.** LC-MS1 chromatographic trace of LC-MS/MS run before and after Fe^3+^-IMAC enrichment. Annotated peaks in the elution fraction correspond to unreacted probe or fragments thereof in the LC-MS run. MS1 peak at red arrow corresponds to unreacted and ether-cleaved phosphonate-PF-06672131 (690.21 and 416.13 *m/z*), similar to the diagnostic ions in Figure 2B. MS1 peak at the blue arrow corresponds to probe with dinitrogen and dimethylamine loss (663.16 *m/z* and 645.15 *m/z*). **B.** PSMs and intensities derived from PF-06672131-bound, phosphorylated (STY) and unmodified peptides in triplicate LC-MS/MS runs of inputs (before enrichment) and elutions (after enrichments). **C.** Bar graph showing the proportions of PSMs derived from PF-06672131-bound, phosphorylated and unmodified peptides in LC-MS/MS runs before and after enrichment. **D.** Bar graph of amount of proteins that have 1 or more PF-06672131-bound sites in intact cells and lysates detected with PhosID-ABPP, showing it is not uncommon to have more than 1 probe-binding site per protein.


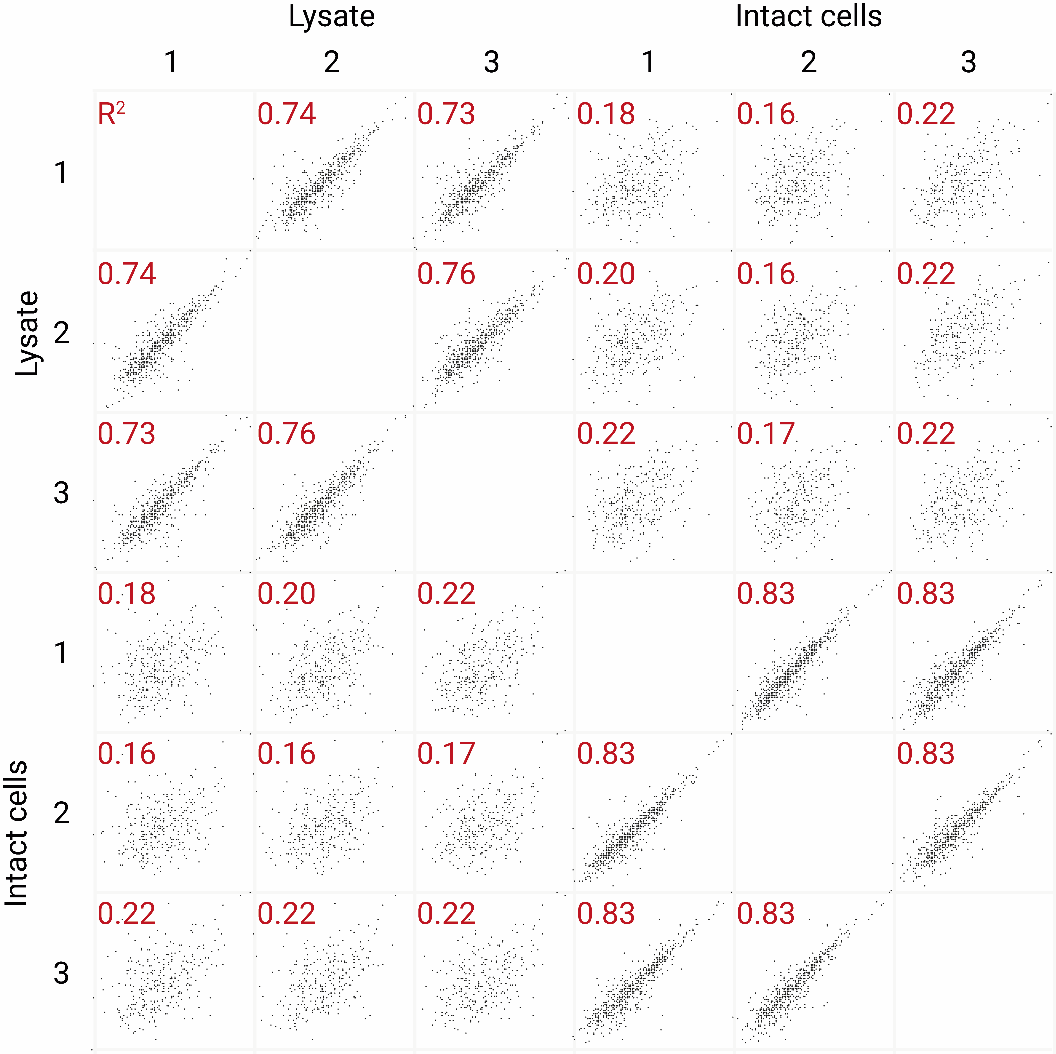


**Supplementary Figure 3**: R-squared correlation of relative abundance of probe-bound peptides detected in LC-MS/MS runs of replicates within lysed- and intact cell-treated samples. Replicates within either lysate or intact cell-based experiments show a high correlation (R^2^>0.73). Low correlation is observed when comparing data from lysates with those obtained from intact cells (R^2^<0.25).

**Supplementary Table 1: I**nteractions between PF-06672131 and RHOA as analyzed by the PLIP web server. Table depicts the type of interaction, the residue on RHOA involved and the distance between the two atoms engaging in the interaction. For hydrogen bonds, the distance from hydrogen atoms and donor atoms to the acceptor atoms are indicated by “H-A” and “D-A” respectively. The angle between de donor, hydrogen and acceptor atoms is also given.

| **Type** | **Residue** | **Distance (Å)** |  |  |
| --- | --- | --- | --- | --- |
| Hydrophobic interaction | LYS18 | 3.86 |  |  |
| Hydrophobic interaction | PHE30 | 3.99 |  |  |
| Hydrophobic interaction | TYR34 | 3.84 |  |  |
| Hydrophobic interaction | VAL35 | 3.97 |  |  |
| Hydrophobic interaction | THR37 | 3.59 |  |  |
| Hydrophobic interaction | LYS118 | 3.25 |  |  |
| Hydrophobic interaction | LYS162 | 3.5 |  |  |
| Salt bridge | ASP59 | 2.93 |  |  |
|  |  |  |  |  |
| **Type** | **Residue** | **Distance H-A (Å)** | **Distance D-A (Å)** | **Donor Angle** |
| Hydrogen bond | GLY17 | 2.27 | 2.86 | 117.37 |
| Hydrogen bond | LYS18 | 3.25 | 3.68 | 108.08 |
| Hydrogen bond | THR19 | 2.92 | 3.68 | 133.42 |
| Hydrogen bond | PRO36 | 2.74 | 3.28 | 113.97 |
